# Complete kidney allograft extrusion through a dehiscent wound following graft thrombosis and immunosuppression withdrawal: a case report

**DOI:** 10.1186/s12882-025-04623-0

**Published:** 2025-11-22

**Authors:** Islam K. Madadov, Dilmurat Akhmetov, Elshad S. Nabiev, Berik G. Rgebayev, Nursultan T. Saduakas, Ernur B. Belgibaev, Bolatbek B. Baimakhanov

**Affiliations:** 1 Department of Urology and Kidney Transplantation, National Scientific Center of Surgery named after A.N. Syzganov, Zheltoksan Street 62, Almaty, 050004 Kazakhstan; 2 National Scientific Center of Surgery named after A.N. Syzganov, Almaty, Kazakhstan

**Keywords:** Kidney transplantation, Graft thrombosis, Wound dehiscence, Allograft extrusion, Surgical complication

## Abstract

**Background:**

Complete extrusion of a transplanted kidney through a dehiscent abdominal wound is an extremely rare postoperative complication. While wound-related issues after kidney transplantation are not uncommon, such an extreme event is rarely documented. We present a case illustrating the clinical challenges and management considerations of this complication.

**Case presentation:**

A 36-year-old woman underwent deceased-donor kidney transplantation. Postoperatively, she developed rapid cardiac arrest, severe neuropathic abdominal pain, and probable Cytomegalovirus (CMV) gastroenteritis. Graft thrombosis was subsequently confirmed, and immunosuppressive therapy was discontinued. Over the following weeks, progressive wound dehiscence with purulent discharge led to complete extrusion of the kidney allograft. An urgent transplant nephrectomy was performed. The patient recovered uneventfully and was discharged on postoperative day four.

**Conclusion:**

This case underscores the importance of vigilant wound monitoring and multidisciplinary management in transplant recipients, particularly after graft failure and immunosuppression withdrawal. Early recognition of wound compromise and timely surgical intervention are critical to preventing adverse outcomes.

**Clinical trial number:**

Not applicable.

## Background

Kidney transplantation is the definitive treatment for patients with end-stage renal disease. Compared to dialysis, it offers superior survival and quality of life [1–3]. Despite its advantages, the procedure is associated with surgical risks. Among them, wound complications influence graft survival and morbidity. Hоwever, these complications are not well explored in the literature. About 4.1% to 15.7% of patients undergoing kidney transplantation have a risk of surgical wound dehiscence [4–9]​. These complications are оften associated with factors such as оbesity, diabetes, hypoalbuminemia, and surgical technical variables.

Although wоund dehiscence has been described, the extrusion of a kidney graft after thrombosis and withdrawal of immunosuppression is exceptionally rare. We fоund no cases reported in large cohort studies. This case contributes to the limited literature оn severe wоund complications after transplant and raises practical questions on monitoring and managing surgical wounds in patients with early graft failure.

## Case presentation

A 36-year-old woman with a history of long-standing type 1 diabetes mellitus (onset at age 13) complicated by diabetic nephropathy, retinopathy, and polyneuropathy progressed to end-stage renal disease. In April 2022, she underwent an open deceased-donor kidney transplantation at an external centre abroad. The graft was placed in the right iliac fossa with end-to-side vascular anastomoses to the external iliac artery and vein, and a ureteroneocystostomy was performed using a standard extravesical technique over a double-J stent. Fascial closure was achieved with a continuous double-layer suture using polydioxanone (PDS 1 − 0). Primary graft function was achieved. Urine output was 2200 mL/24 h on POD 0–1 and 3000 mL/24 h on POD 1–2. Serum creatinine declined from 635 µmol/L (POD 0) to 370 µmol/L (POD 1) and 220 µmol/L (POD 2) (65.4% decrease by POD 2). No dialysis was required during the first postoperative week. Early duplex ultrasound (POD [1, 2]) demonstrated preserved graft perfusion (RI [0.65–0.72]). After transplant, CMV PCR became positive and the patient was treated with valganciclovir 900 mg twice daily; no viral load measurements were available. A repeat PCR one month later was negative. There was no fever and no leukocytosis documented. The patient reported irregular daily use of NSAIDs (ketorolac, diclofenac, and ibuprofen), occasionally in combination. The patient had acute and persistent abdominal pain in the early postoperative period. Clinical suspicion was in favour of colitis and gastroenteritis linked to CMV. Although her symptoms were only partially alleviated, she was treated with analgesics, non-steroidal anti-inflammatory drugs (NSAIDs), and antispasmodics. By week 4 post-transplant, renal function remained stable (serum creatinine 120 µmol/L, estimated GFR 52 mL/min/1.73 m²).

The index transplantation was performed abroad, and post-discharge she was followed by a private nephrologist; ambulatory glucose logs and titration records were unavailable. In May 2022 laboratory testing recorded HbA1c 8.88% and serum glucose 14.74 mmol/L. Haemoglobin was 99 g/L in Nutritional indices showed BMI 19.3 kg/m², total protein 53.5 g/L, and albumin 38.2 g/L.

In May 2022, approximately one month after transplantation, she experienced a sudden cardiac arrest and was successfully resuscitated. Subsequent imaging demonstrated renal allograft arterial thrombosis with absent perfusion, consistent with secondary loss of graft function. Immunosuppressive therapy was discontinued after non-viability of the graft had been established. Her abdominal pain subsequently improved. The aetiology of the abdominal pain remained uncertain. Initially, CMV gastritis/duodenitis/colitis was suspected clinically, but this was not confirmed. The pain subsided after withdrawal of maintenance immunosuppression, suggesting a possible medication-related component. The patient reported that the surgical wound in the right lower quadrant remained fully closed and clinically stable until early July 2022. However, she noticed purulent discharge and progressive dehiscence on 8 July 2022.

**Fig. 1:**
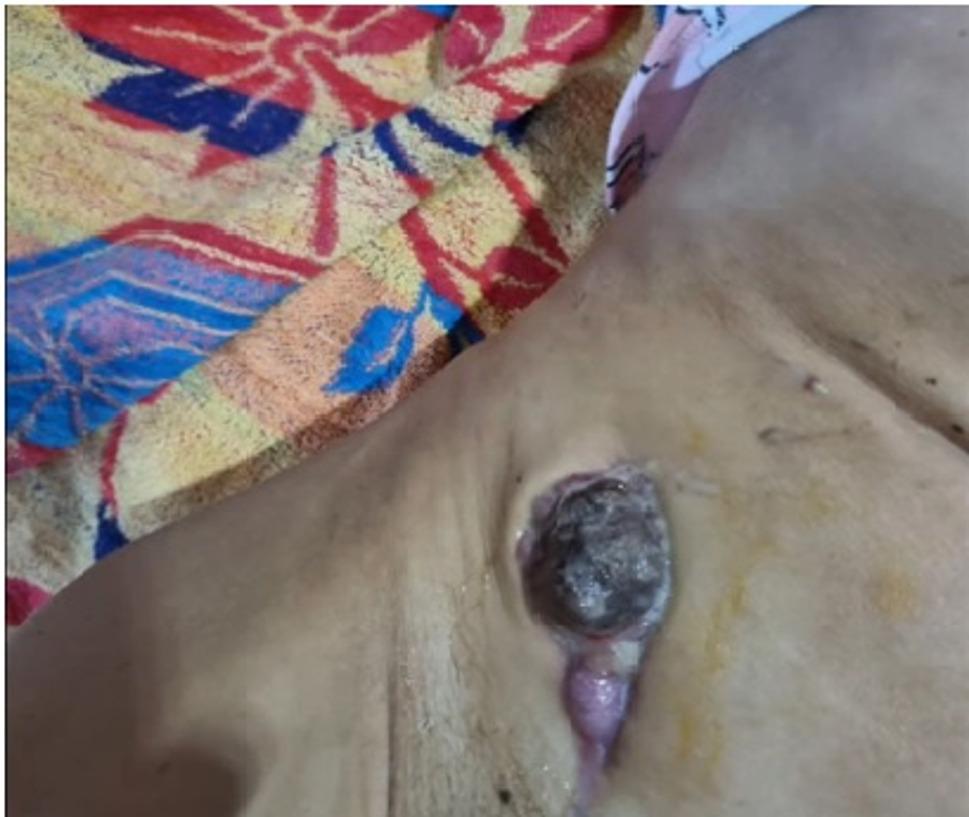
External view of the transplanted kidney partially visible through the dehiscent abdominal wound (19 July 2022)

**Fig. 2:**
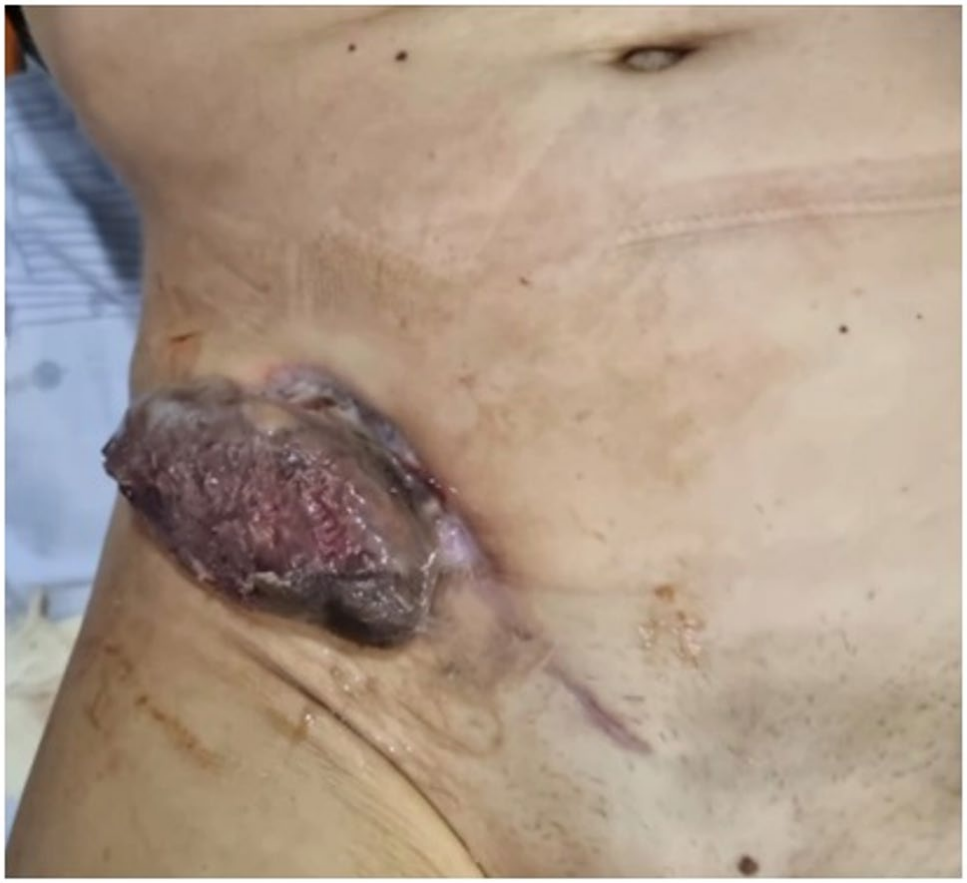
Complete extrusion of the renal allograft through the surgical wound, resting externally on the abdominal wall (20 July 2022)

Over the next few days, the drainage volume steadily dropped. On 19 July, the top pole of the kidney allograft became externally apparent and wound dehiscence was noted (Fig. 1). Immediately following a standard haemodialysis session on 20 July, the transplanted kidney completely protruded through the abdominal incision (Fig. 2). The patient attended our transplant centre the next day (21 July 2022) for a planned admission.

The patient’s vital signs were stable at admission. The renal transplant was fully externalized and only attached to the patient through the ureter. The patient did not have a high temperature or haemodynamic instability, although she complained of mild abdominal pain. We report the data we do have at readmission to our hospital: laboratory investigations revealed hyperglycaemia (14.6 mmol/L), increased serum creatinine (296 µmol/L), and anaemia (haemoglobin 66 g/L). Her HbA1c was at 9.49%; graft artery thrombosis and significant ascites were confirmed by a previous ultrasonography.

**Fig. 3:**
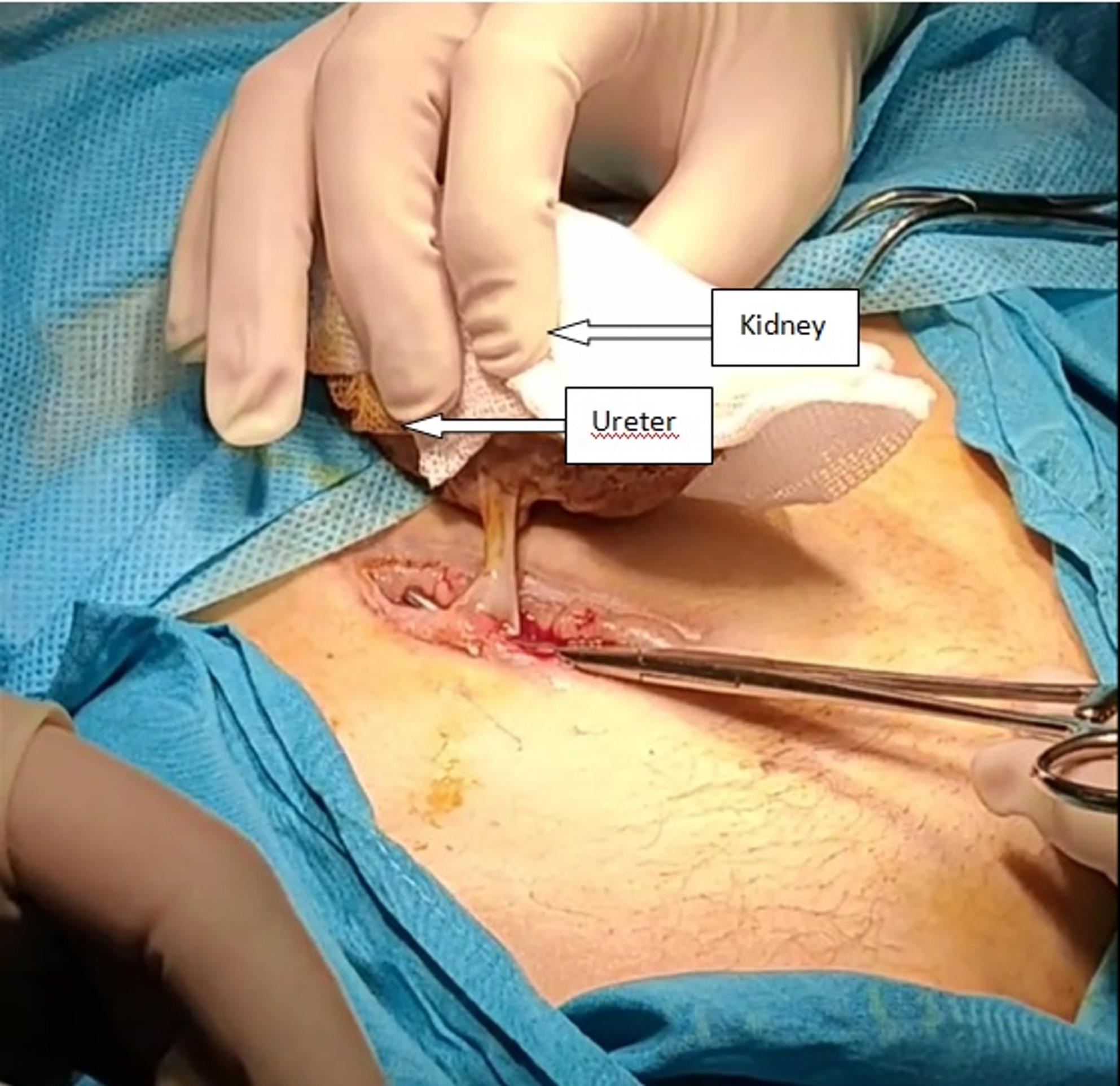
Intraoperative image of the devascularized kidney graft attached solely via the ureter before explantation

We performed surgical exploration on 22 July (Fig. 3). Complete autolysis of the vascular pedicle was verified intraoperatively. The kidney graft was only attached to the patient through the ureter. After dividing and ligating the ureter, the non-viable graft was extracted. The wound cavity was dressed and irrigated. The procedure was uneventful.

**Fig. 4:**
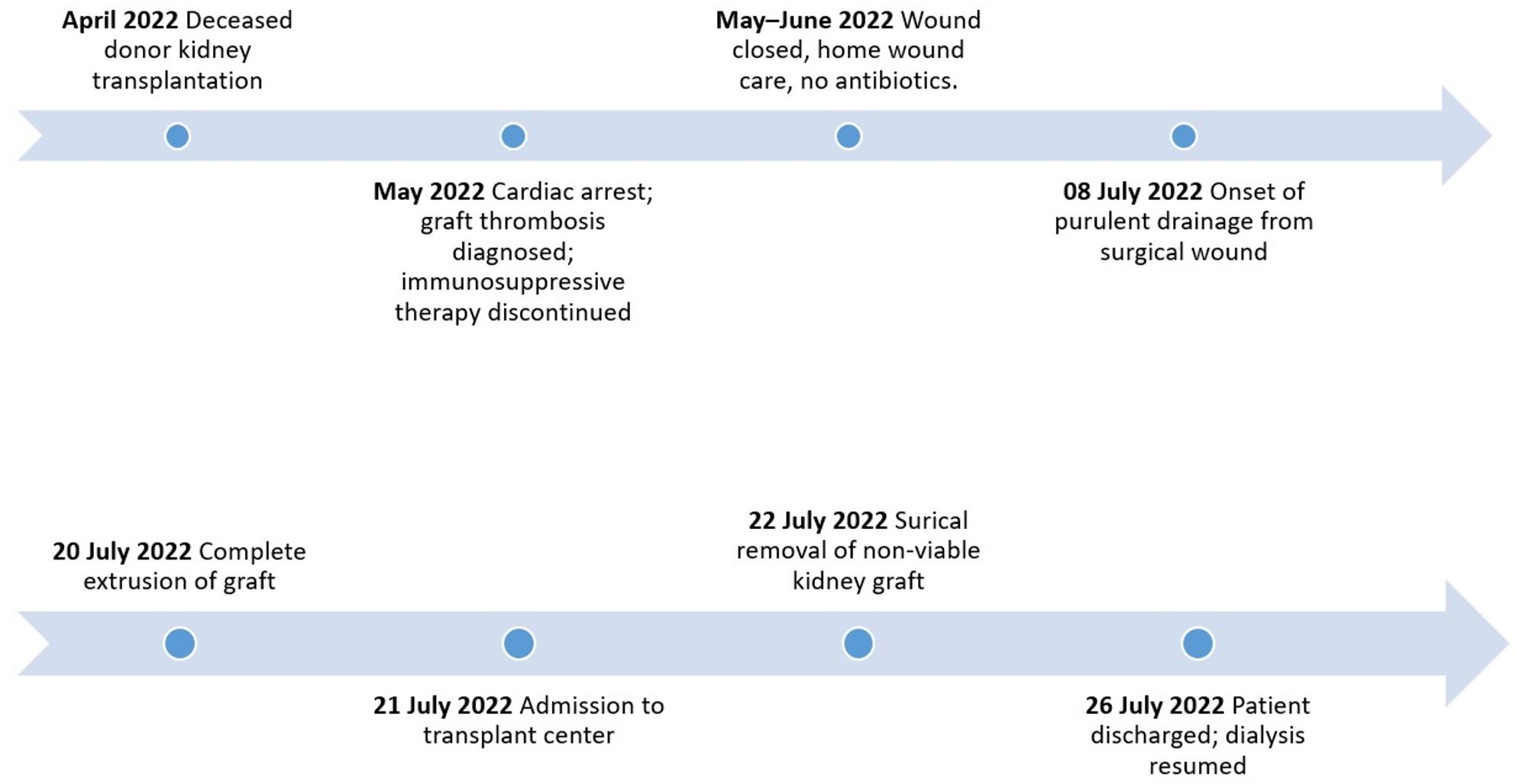
Thetimeline

The patient completely recovered following surgery. Within 24 h, she was moved from the intensive care unit to a general ward, and on the fourth postoperative day, she was discharged in stable condition. She is presently being evaluated for a potential future retransplant and has resumed her scheduled haemodialysis. The timeline of the patient’s treatment during hospitalisationis shown in Fig. 4.

### Discussion and conclusions

Complete extrusion of a renal allograft through a dehiscent surgical wound is an exceedingly rare postoperative complication in kidney transplantation. To our knowledge, no previously published reports have documented a case of complete renal allograft extrusion through a dehiscent surgical wound, making this an exceptionally rare and unreported complication. In this case, the extrusion occurred in the context of vascular graft thrombosis and subsequent withdrawal of immunosuppression—an unusual but significant combination that likely contributed to progressive wound failure.

After a kidney transplant, wound problems are somewhat common and usually include partial wound dehiscence, superficial infections, or lymphoceles [10, 11]. Conservative treatment or minimal surgical correction can often manage these problems. However, complete allograft extrusion through the abdominal wall is difficult to manage both clinically and surgically and is not likely to occur within the anticipated postoperative period.

Several factors likely contributed to this event. First, after cardiac arrest, the patient had graft thrombosis, which required an immediate discontinuation of immunosuppression. Abrupt discontinuation of immunosuppressive therapy following graft failure may lead to an immune rebound—particularly involving T lymphocytes—triggering local inflammation, cytokine release, and Major histocompatibility complex(MHC)upregulation [12–15]. Although Immune Reconstitution Inflammatory Syndrome (IRIS) -like phenomena have not been documented in transplant recipients without HIV infection, to our knowledge, such immune activation could theoretically exacerbate tissue injury and impede wound healing. In the presence of residual viable graft tissue or pre-existing inflammation, this may contribute to rare but severe complications such as graft extrusion. Second, the patient had been using various NSAIDs (including ketorolac, diclofenac, and ibuprofen) on a prolonged and uncontrolled basis from the time of transplantation in April 2022 until the discontinuation of immunosuppression. These medications were taken repeatedly and on a rotation basis to manage chronic neuropathic abdominal pain. Prolonged NSAID use is known to impair soft tissue and wound healing by inhibiting cyclooxygenase activity. This reduces prostaglandin-mediated angiogenesis, fibroblast proliferation, and collagen synthesis [16–19]. In this case, NSAID-induced suppression of inflammatory and reparative pathways may have contributed to delayed wound healing and subsequent dehiscence, especially in the presence of diabetes mellitus. Third, CMV colitis was clinically suspected but not confirmed due to the absence of endoscopic or histopathological verification, representing a limitation of this investigation. Nevertheless, CMV infection in transplant recipients can exacerbate systemic inflammation through cytokine release, impair immune regulation, and contribute to gastrointestinal dysmotility, which may indirectly compromise wound healing and tissue integrity [20, 21].

Poorly controlled diabetes is a well-known risk factor for impaired wound healing and surgical site complications, including dehiscence [22–24]. Mechanistically, sustained hyperglycaemia impairs neutrophil chemotaxis and phagocytosis, alters macrophage polarisation, disrupts collagen synthesis and cross-linking, attenuates VEGF-mediated angiogenesis, compromises microvascular perfusion, and augments oxidative stress, collectively delaying acquisition of tensile strength and wound closure. Taken together, the longitudinal HbA1c profile and these well-described pathways support diabetes as a material predisposing factor in the cascade culminating in wound dehiscence and extrusion. In this case hyperglycaemia likely acted in concert with additional adverse modifiers present at the time: irregular daily NSAID use (ketorolac, diclofenac, and ibuprofen, sometimes in combination), which can blunt prostaglandin-dependent angiogenesis and fibroblast activity; marginal nutritional reserve (BMI 19.3 kg/m², total protein 53.5 g/L, albumin 38.2 g/L); and intercurrent CMV infection (PCR-positive post-transplant, treated with valganciclovir; later PCR-negative), which may amplify systemic inflammatory tone even in the absence of fever or leucocytosis. Acute physiological stress around clinical deterioration may also have amplified insulin resistance, further elevating glucose at presentation; nonetheless, the chronically high HbA1c values indicate long-term suboptimal control as the dominant backdrop. While HbA1c 9.49% argues for chronically inadequate glycaemic control, we acknowledge that acute physiological stress can precipitate transient insulin resistance, and therefore the observed hyperglycaemia likely reflects a combination of poor long-term control and stress hyperglycaemia. Peri-transplant and early postoperative glycaemic records were unavailable because the index transplantation was performed abroad and subsequent follow-up occurred in a private clinic, with discharge documents and ambulatory logs not retrievable. This precludes granular reconstruction of glycaemic trajectories and insulin titration. Despite these constraints, the available data, the patient’s diabetic phenotype, and biological plausibility together support diabetes as a central contributor to impaired wound healing in this case.

Collectively, these factors created a vulnerable wound environment in which even modest mechanical stress could precipitate full-thickness dehiscence and graft extrusion. Whether more proactive wound care or an earlier surgical re-evaluation may have prevented this outcome remains unclear. However, this case highlights the importance of careful postoperative monitoring, especially for patients who have overlapping risk factors or early graft failure.

In the present case, after extrusion, the clinical reaction was quick and efficient. The patient’s devitalized graft was successfully explanted without any surgical difficulties. Her uneventful recovery highlights the importance of coordinated surgical treatment, even in emergency situations. However, the case raises a number of points worth considering. Although clinically warranted, the timing of immunosuppressive discontinuation may have worsened tissue injury, and the delayed advancement of wound dehiscence may have lacked obvious early indicators.

In the setting of confirmed non-viable allograft (arterial thrombosis with absent perfusion), immediate withdrawal of maintenance immunosuppression is clinically appropriate to limit the risks of infection, impaired wound healing, and metabolic complications. Close post-withdrawal follow-up by transplant surgery and transplant nephrology is essential, with attention to wound status, glycaemic control, and signs of systemic inflammation. Detailed tapering information (e.g., steroid taper) was not available in the medical record and is acknowledged as a limitation.

For high-risk recipients with long-standing type 1 diabetes, early post-transplant care and any period following changes in immunosuppression should incorporate structured capillary glucose monitoring with insulin-based adjustment to maintain safe, moderate targets, alongside nutrition optimisation and avoidance of prolonged NSAIDs. Scheduled wound assessments with a low threshold for earlier review (e.g., with new drainage, erythema, or pain) are advisable. Where HbA1c interpretation is potentially confounded by anaemia or transfusion, complementary indices (fructosamine or glycated albumin) and objective glucose profiles can refine assessment of true glycaemic exposure.

Chronic abdominal pain in transplant recipients may arise from gastrointestinal inflammation, postoperative neuralgia, or medication-related effects. In this case, CMV gastrointestinal disease was suspected but unconfirmed, and the resolution of pain following withdrawal of maintenance immunosuppression raises the possibility of a drug-related contribution. We therefore do not attribute the pain to rejection; at the time of assessment, laboratory indices and imaging showed no evidence of allograft rejection or dysfunction, and subsequent graft loss was accounted for by arterial thrombosis with absent perfusion. Nevertheless, in the absence of graft biopsy, rejection cannot be definitively excluded and this remains a limitation.

After discontinuation of maintenance immunosuppression for a non-viable allograft, transplant nephrology should co-lead follow-up with surgery. Priorities: early review within 1–2 weeks, then 2-4-weekly as needed; wound checks at each visit with a low threshold for surgical reassessment; optimisation of glycaemic control and correction of anaemia/nutritional deficits; infection surveillance (including CMV when relevant); analgesia stewardship with avoidance of prolonged NSAIDs; targeted imaging only if collections/bleeding are suspected; and timely consideration of completion nephrectomy if ongoing risk persists. Provide patient education on warning signs and escalation.

This case highlights the risks of transplant tourism. When patients undergo transplantation abroad, they may not receive coordinated, continuous care, and the original transplant team may not be involved in postoperative follow-up. Poor communication between centers, limited access to perioperative records, and the lack of standardized wound and metabolic monitoring can all lead to complications that might otherwise be prevented. In Kazakhstan, the shortage of deceased-donor kidneys and the limited pool of suitable living donors have pushed some patients to seek transplants in other countries. This pattern reflects a broader problem of organ scarcity and points to the need to: strengthen national deceased-donor programs, ensure continuity of post-transplant care, and develop international communication protocols for patients transplanted abroad. Improving international cooperation and ensuring long-term, multidisciplinary follow-up are key to enhancing patient safety and graft survival.

This case also raises broader implications for post-transplant care. Structured wound monitoring protocols may be especially important in patients with gastrointestinal symptoms, chronic pain requiring anti-inflammatory medications, or abrupt immunologic transitions. In our view, symptomatic recipients are best seen within 1–2 weeks, with reassessment every 2–4 weeks until stable. Follow-up should emphasise the trajectory and localisation of pain, a focused examination, and selective testing (renal profile, full blood count, and—when inflammatory features are suspected—CRP). Doppler ultrasonography is, in our experience, most informative when red flags emerge (progressive pain, fever, increasing drainage or erythema, a palpable/fluctuant mass, or falling haemoglobin). We favour a low threshold for urgent surgical review, avoidance of prolonged NSAIDs, and discussion of completion nephrectomy where infection risk persists, collections recur, or pain remains refractory. Additionally, interdisciplinary management involving experts in transplant surgery, nephrology, infectious diseases, and pain medicine can be critical in preventing rare but critical complications. 

## Data Availability

All data generated or analyzed during this study are included in this published article. Additional supporting data (e.g., de-identified medical records, imaging) are available from the corresponding author upon reasonable request.

## Abbreviations

AChT: Activated partial thromboplastin time
BMI: Body mass index
CMV: Cytomegalovirus
CRP: C-reactive protein
CT: Computed tomography
DIC: Disseminated intravascular coagulation
ESRD: End-stage renal disease
HbA1c: Glycated hemoglobin
ICU: Intensive care unit
intensive care unit: Immune reconstitution inflammatory syndrome
NSAIDs: Non-steroidal anti-inflammatory drugs
POD: Postoperative day
RAI: Rejection activity index
SWD: Surgical wound dehiscence
TP: Transplanted kidney
US: Ultrasound
